# Synergistic Flame Retardant Effect of Barium Phytate and Intumescent Flame Retardant for Epoxy Resin

**DOI:** 10.3390/polym13172900

**Published:** 2021-08-28

**Authors:** Linyuan Wang, Yue Wei, Hongbo Deng, Ruiqi Lyu, Jiajie Zhu, Yabing Yang

**Affiliations:** 1 School of Chemistry and Chemical Engineering, Southwest Petroleum University, Chengdu 610500, China; ztszd1015@163.com (Y.W.); cejsomi@163.com (H.D.); Lrainbowchser77@163.com (R.L.); lhjksj@163.com (J.Z.); 2 Petroleum Engineering Design Co., Ltd., China Petrochemical Corporation, Dongying 257026, China; yybjennie@163.com

**Keywords:** intumescent flame retardant, barium phytate, epoxy resin, synergistic effect

## Abstract

Recently, widespread concern has been aroused on environmentally friendly materials. In this article, barium phytate (Pa-Ba) was prepared by the reaction of phytic acid with barium carbonate in deionized water, which was used to blend with intumescent flame retardant (IFR) as a flame retardant and was added to epoxy resin (EP). Afterward, the chemical structure and thermal stability of Pa-Ba were characterized by Fourier transform infrared (FTIR) spectroscopy and thermogravimetric analysis (TGA), respectively. On this basis, the flammability and flame retardancy of EP composites were researched. It is shown that EP/14IFR/2Ba composite has the highest limiting oxygen index (LOI) value of 30.7%. Moreover, the peak heat release rate (PHRR) of EP/14IFR/2Ba decreases by 69.13% compared with pure EP. SEM and Raman spectra reveal the carbonization quality of EP/14IFR/2Ba is better than that of other composites. The results prove that Pa-Ba can cooperate with IFR to improve the flame retardancy of EP, reducing the addition amount of IFR in EP, thus expanding the application range of EP. In conclusion, adding Pa-Ba to IFR is a more environmentally friendly and efficient method compared with others.

## 1. Introduction

In recent years, safety and environment protection requirements have become higher and higher for many materials used in industry and daily life. Epoxy resin (EP) plays a significant role in mechanical properties, electrical insulation, heat resistance, corrosion resistance and so on, thus becoming one of the most indispensable resins. It is extensively used in coatings, electronic and electrical industries, handicrafts, and photoelectric industries. However, EP is composed of chains of hydrocarbon with high flammability, which will produce comparatively great toxicity in the process of combustion. Therefore, there is an urgent need to improve the flame retardancy of EP [1,2,3,4,5].

In order to improve the flame retardancy of EP [6,7,8,9], there are several commonly used methods, such as surface modification [10], superrefining [11,12,13], complex cooperation [14,15], and cross-linking. A lot of research works have been carried out, and the direct addition of flame retardants has the advantages of convenience and economy, thus becoming the most chosen approach. Halogen flame retardant has advantages of high flame retardant efficiency [16], low dosage and good compatibility with materials, however, a large amount of smoke and poisonous and corrosive gases, such as dioxins, will be produced during combustion, causing great harm to the environment. Moreover, metal hydroxide is also an available flame retardant synergist, which is non-toxic and has good stability, while plenty of additive amounts and poor flowability will reduce the mechanical properties of materials. Therefore, we consider IFR as an environmentally friendly flame retardant [17], which is halogen-free and has low smoke. The most familiar and commercial IFR system is ammonium polyphosphate (APP) [18] and pentaerythritol (PER) [19,20,21], but its flame retardant efficiency is not high enough for EP. In order to decrease the additive amount and improve the flame-resistant efficiency, more and more attention has been paid to adding synergistic agents, a productive approach to improve flame retardancy.

At present, with the development trend of social environment friendliness, wide attention has been paid to bio-based flame retardants. Some natural materials have been used as flame retardants, such as deoxyribonucleic acid [22], gluten [23,24,25], starch [26], tea saponin [27,28,29], chitosan [30], and phytic acid (Pa) [31,32,33]. Pa consists of six phosphate groups, which is beneficial to the electrochemical stripping and makes the system have high flame retardancy. The recent researches reveal that Pa has been used as a bio-based flame retardant due to its biodegradability, compatibility, high phosphorus content (28 wt%) and non-toxic properties, and is able to improve the flame retardancy of ethylene-vinyl acetate [34], polypropylene [35,36,37], polylactic acid [38], cotton fabrics [39], and epoxy resin. Jaime C.Grunlan et al. first used Pa as a flame retardant [40]. They deposited Pa and chitosan on the surface of cotton fabrics to prepare completely renewable and environment-friendly electrolyte films. The PHRR of the coated fabric with pH 4 decreased by 60% by micro-combustion calorimetry. Wang et al. combined Pa with nitrogen and silicon compounds to improve the flame retardancy of polylactic acid [28]. They deposited nitrogen-containing silane hybrid and Pa coating on polylactic acid by layer-by-layer self-assembly technology, which significantly improved the fire resistance. However, the direct incorporation of Pa with acidity into EP may reduce its thermal stability and mechanical properties.

It is widely known that metal compounds have the ability to dehydrogenate and are excellent synergistic carbonization agents, especially barium compounds. The barium ion has been proven to effectively promote the reaction between APP and PER and enhance its flame retardant efficiency. Additionally, the reaction of cationic Ba^2+^ with the anion of Pa may have a dual synergistic effect, thus enhancing the flame retardancy of the EP/IFR system. It is found that even a small amount of barium compounds could markedly increase the LOI of EP, concurrently improving the compactness of the carbon layer after combustion. At the same time, Pa can chelate with zinc, cobalt, nickel, barium, aluminum and other metal ions to form the corresponding compound, named phytate [41,42]. Hu et al. applied the graphene functionalized by ferric phytate to the flame retardant study of EP [33], which remarkably reduced the HRR of EP materials, forming a highly protective carbon layer and relatively lowing yield of CO. Phytate, assigned to a green and environment-friendly bio-based material and used as the antioxidant of oil, preservative of food and fruit, and corrosion inhibitor of metal materials, can be extensively applied in food, petrochemical and medical fields, thus having a good prospect of development and application.

Currently, there are few studies on adding more environmentally friendly phytate as a synergistic agent in EP/IFR system, mostly adding silicon flame retardant or surface modification. However, these are not environmentally friendly compared with phytate, and the flame retardant effect is not efficient enough. APP in IFR system is derived from non-renewable phosphate rock resources, and the main component of Pa-Ba is renewable bio-based material Pa. Replacing non-renewable flame retardants with renewable flame retardants can effectively alleviate the problem that phosphate rock resources will eventually be exhausted, which is the general trend under the current environment. In this article, barium phytate (Pa-Ba) was prepared from phytic acid (Pa) and barium carbonate, the synthesized Pa-Ba and IFR were then added to EP by blending to prepare EP composites. The thermal stability was investigated by thermogravimetric analysis (TGA), and the flame retardancy was analyzed by limiting oxygen index and cone calorimeter. Therefore, phytic acid, a bio-based raw material with flame retardancy, was selected to combine inorganic metal ions with a catalytic effect to prepare a synergist to improve the flame retardant efficiency and reduce the adjunction amount of IFR in EP. This method can not only improve the effect of a high content of flame retardant on EP matrix, but also become more economical because of the reduction in the amount of APP originating from non-renewable resources and the increase in Pa-Ba and make EP widely used in various fields.

## 2. Materials and Methods

### 2.1. Materials

Epoxy resin (EP-51) and APP (*n* > 1500) were purchased from Beijing Hanlongda Technology Development Co., Ltd. (Beijing, China). Phytic acid (70% aqueous solution) was purchased from Chengdu Huaxia Chemical Reagent Co., Ltd. (Chengdu, China), pentaerythritol (PER) was provided by Qingdao Keer Science and Trade Co., Ltd. (Qingdao, China), barium carbonate was bought from Shanghai Gaoxin Chemical Glass Instrument Co., Ltd. (Shanghai, China), and polyamide curing agent (PA651) was purchased from Beijing Mairuida Technology Co., Ltd. (Beijing, China).

### 2.2. Preparation of Pa-Ba

**Scheme 1:**
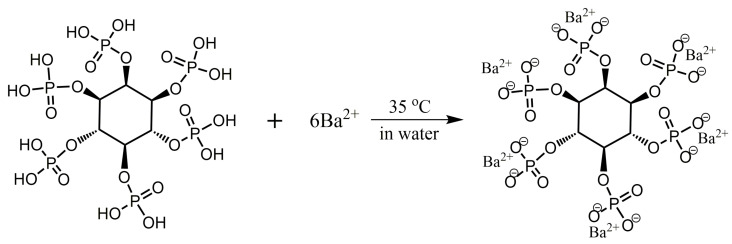
Synthesis route of Pa-Ba.

The synthesis diagram of Pa-Ba prepared from Pa and barium carbonate is shown in Scheme 1. First, 11.96 g (0.06 mol) BaCO_3_ was suspended in 100 mL deionized water under 35 °C, and 9.43 g (0.01 mol) Pa (70% aqueous solution) was dissolved into 50 mL of deionized water. After they dissolved completely, the Pa solution was placed in a constant pressure droplet funnel, which was added to the BaCO_3_ suspension at the rate of 5 drops 10 s within 30 min, with mechanical stirring. The reaction was kept at a constant temperature for 3 h, until there was no precipitation. Afterwards, the white precipitation yielded was filtered and rinsed with deionized water no less than 5 times until PH was equal to 7. Finally, the product was dried at 75 °C for 10 h, and a white powder, namely Pa-Ba, was obtained. The yield of Pa-Ba is about 85.88%.

### 2.3. Preparation of EP Composites

The composition of EP is shown in Table 1. At the very beginning, EP systems were slowly stirred for 30 min under 75 °C after the participation of IFR and Pa-Ba, so that the flame retardant was uniformly dispersed in the epoxy resin. The curing agent PA651 (the mass ratio of EP to PA651 was 3:1) was added into EP composites, stirring until the mixture was uniform. Afterwards, the blends were dried in a vacuum oven at 100 °C for 3 h and injected slowly into the mold which was preheated in 10 min and cured in a constant temperature drying box by the curing system of 110 °C/3 h + 130 °C/3 h + 150 °C/2 h. The EP composites were obtained after natural cooling.

### 2.4. Measurements

The FTIR was performed with the Thermo Fisher Nicolet ls10 spectrometer (Beijing Ruili Analytical Instrument Co., Ltd., Beijing, China) by recording the frequency of 16 scans, and the region was 400–4000 cm^−1^. The sample functional groups were tested by KBr pressing method.

Thermogravimetric analysis (TGA) (Netzsch, Germany) was responded under the heating rate of 20 °C/min from 40–800 °C in N_2_ atmosphere.

The LOI tests were performed on a DRK304B oxygen index tester (Kunshan Modisco Combustion Technology Instrument Co., Ltd., Kunshan, China). The dimension of epoxy splines was 130.0 × 6.5 × 3.0 mm^3^.

Based on the ISO 5660-1:2002, cone calorimeter was utilized with the heat flux 35 kW/m^2^, the dimension of epoxy splines was 100 × 100 × 3 mm^3^.

The synthesized samples and the residual chars after burning were observed by ZEISS EV0 MA15 scanning electron microscope(Carl Zeiss, Germany).

With the laser wavelength 532 nm, the Raman spectra were recorded in the range 200–2000 cm^−1^ by using the Thermo Fisher Dxr2xi Confocal Raman spectrometer (RENISHAW plc, Wotton-under-Edge, UK).

## 3. Results and Discussion

### 3.1. Characterization of Pa-Ba

**Figure 1:**
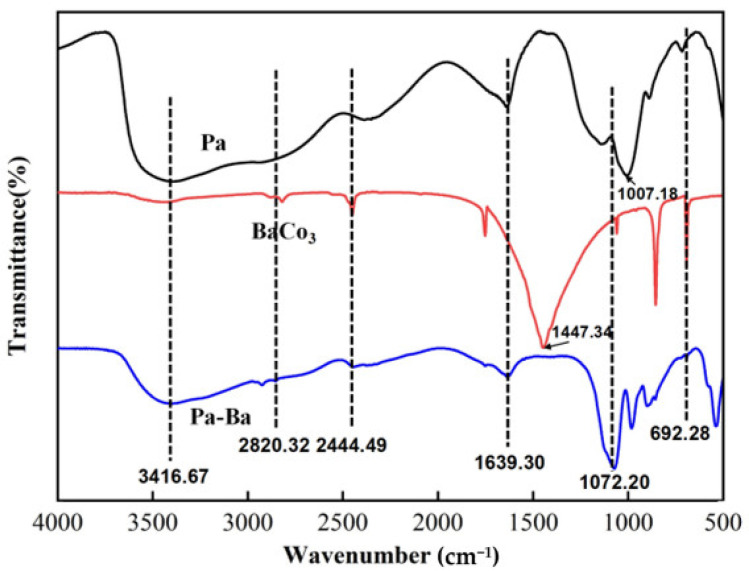
FTIR spectra of Pa, BaCO_3_, and Pa-Ba.

In accordance with Scheme 1, Pa-Ba was produced. Figure 1 indicates the FTIR spectra of Pa, BaCO_3_ and Pa-Ba [43]. As shown in Pa, 3416.67 cm^−1^ is associated with the O–H absorption of H_2_O, the appearance of the O–P–O telescopic vibration is shown in 1639.30 cm^−1^, and CH_2_ meets the conditions of characteristic vibration peak at 2846–2942 cm^−1^, the position of 2820.32 cm^−1^ is attributable to the vibration shrinkage range of P–OH. It is found that BaCO_3_ shows the absorption peak of C=O at 1447.34 cm^−1^, 692.28 cm^−1^ is the characteristic band of barium salt. As for Pa-Ba, some characteristic peaks are observed from barium salt and Pa, for example, the peaks at 692.28 cm^−1^, just as the same with BaCO_3_ and the absorption for (PO_3_)^2−^ at 1007.18 cm^−1^ shifts to 1072.20 cm^−1^, which means the interaction between ions is changed. Moreover, the vibration of P–OH at 2820.32 cm^−1^ and the absorption peak of C=O at 1447.34 cm^−1^ both disappeared. The results above suggested that Pa-Ba was synthesized.

**Figure 2:**
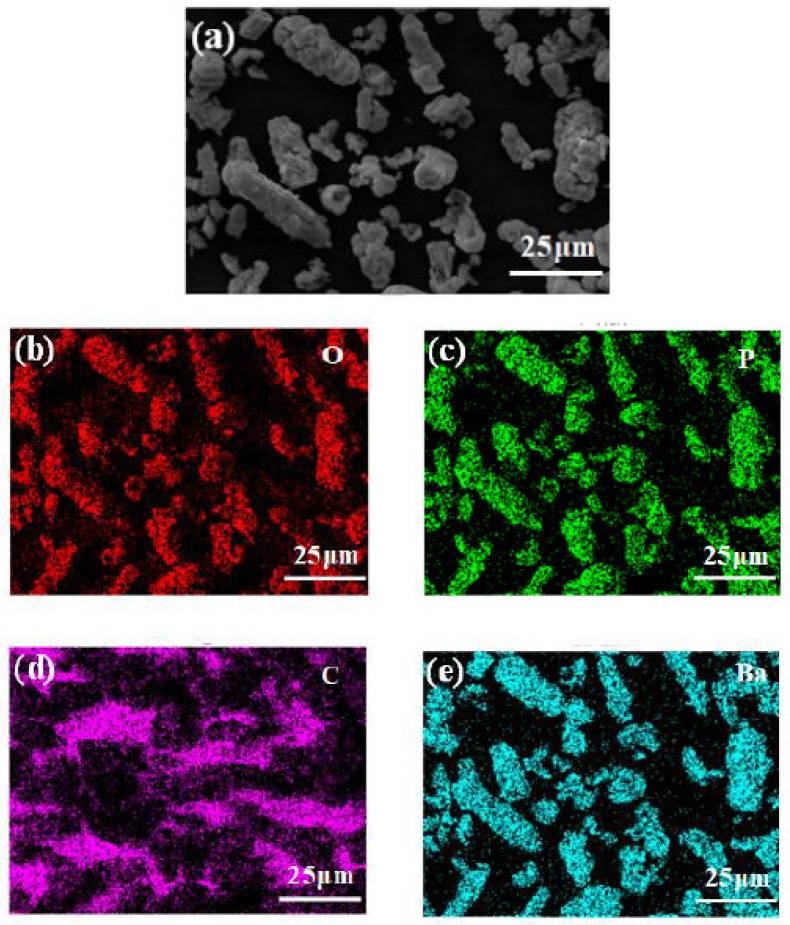
(**a**) SEM of Pa-Ba and (**b**–**e**) EDS spectrum of four main elements of Pa-Ba.

Barium phytate was characterized by Scanning electron microscopy (SEM) and energy-dispersive spectroscopy (EDS) as shown in Figure 2 [44]. Figure 2a shows the SEM morphology of Pa-Ba which has an irregular granular shape. The EDS data of Pa-Ba are depicted in Figure 2b–e, which demonstrate the composition and distribution of main elements in Pa-Ba. It is evident that there are four main elements oxygen (O), phosphorus (P), carbon (C), and barium (Ba) in Pa-Ba. In addition, the four main elements are distributed homogeneously, which further confirms that Pa-Ba was synthesized.

### 3.2. Thermal Stability

**Figure 3:**
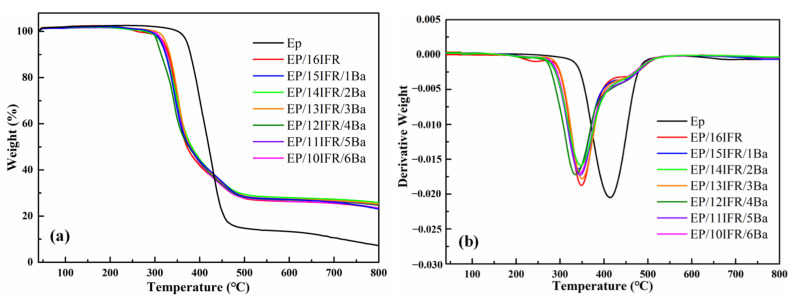
(**a**) TGA and (**b**) DTG curves of pure EP and EP composites in nitrogen.

To investigate the reactions among Pa-Ba, EP and IFR, their thermal decomposition behaviors are researched using TGA in the N_2_ atmosphere as displayed in Figure 3. The details are shown in Table 2.

The degradation of pure EP can be divided into the following two stages. The first stage occurs from 350 to 500 °C, which is also the main thermal degradation stage of EP. The degradation of EP can release a large amount of heat and produce CO, CO_2_, CH_4_ and other thermal degradation gases. In the second stage, after 500 °C, EP continuously degrades and carbonizes to form a carbon layer. With the addition of IFR and Pa-Ba, the carbon layer increases ulteriorly, which can better isolate the exchange of heat and gas and prevent combustion. Furthermore, there are some exothermic and endothermic events in TGA. The endothermic reaction occurs when APP is heated to 300–330 °C, and the side-chain structure decomposes and removes part of the amino groups to form hydroxyl groups. The exothermic reaction occurs after the thermal decomposition of APP, which reacts with PER to form a homogeneous carbon layer. The char formation of IFR is instrumental in restraining the combustion of EP composites, thus improving thermal stability. In addition, Pa-Ba reacts with IFR exothermically at 350–600 °C to form a more stable carbon layer.

As exhibited in Figure 3a and Table 2, the initial decomposition temperature (T_5 wt%_) of pure EP occurs at 372 °C, and there is obvious weightlessness at 300–500 °C. After adding Pa-Ba and IFR, the weightlessness stage of EP composites is identical to that of pure EP. However, the T_5 wt%_ of EP composites decreases in different degrees, for example, the T_5 wt%_ for EP/16IFR and EP/14IFR/2Ba decreases from 372 °C to 320 °C and 313 °C, respectively, which reveals that IFR and Pa-Ba advance the decomposition of EP [43]. The residue of EP/IFR with 23.27 wt% at 800 °C enhances remarkably in comparison with that of pure EP with 7.25 wt%, which means that a stable carbon layer was generated by the reaction of APP and PER. After adding Pa-Ba, the residue of EP composites increases further, especially, EP/14IFR/2Ba composite has the highest residue of 25.90 wt%.

From the DTG curves in Figure 3b, the maximum weight loss rate and the T_max_ of EP composites both show downward trends. EP/IFR/2Ba composite has the lowest maximum thermal decomposition rate, corresponding to a temperature of 347 °C, which is 67 °C lower than that of pure EP. It is proved that IFR and Pa-Ba can reduce the T_max_ of EP, generating more residues and forming more stable carbon layers.

Lower initial decomposition temperature, the reduction in the T_max_ and the increase in the residues demonstrate that Pa-Ba can be used as a carbon source, which can protect the matrix degradation, having the effect of heat insulation and oxygen insulation, thus producing more stable carbon layers.

### 3.3. Flame Retardancy

As demonstrated in Table 3, the pure EP shows high flammability when the LOI reaches 19.1%. Obviously, the adjunction of IFR increases the LOI of EP from 19.1% to 24.3%, which availably improves the flame retardancy of EP, revealing IFR can be considered as a productive flame retardant. With Pa-Ba added into EP composites, the LOI values exhibit a trend of increasing at first and then decreasing, especially, EP/14IFR/2Ba composite has the highest LOI reaching 30.7%. The results demonstrate that Pa-Ba can increase the flame retardant efficiency of IFR in EP, thus making EP widely used in various fields. This section may be divided into subheadings. It should provide a concise and precise description of the experimental results, their interpretation, as well as the experimental conclusions that can be drawn.

Cone calorimeter test (CCT) is adopted to test the flammability of polymer materials, the combustion behavior in a fire can be assessed in accordance with the experimental data [41]. There are many combustion parameters of combustible materials obtained by CCT in a fire. The flame retardancy in an actual fire can be evaluated by heat release rate (HRR), peak heat release rate (PHRR), and total heat release (THR). Meanwhile, the smoke suppression properties can be estimated by smoke production rate (SPR), peak smoke production rate (PSPR), total smoke production (TSP).

**Figure 4:**
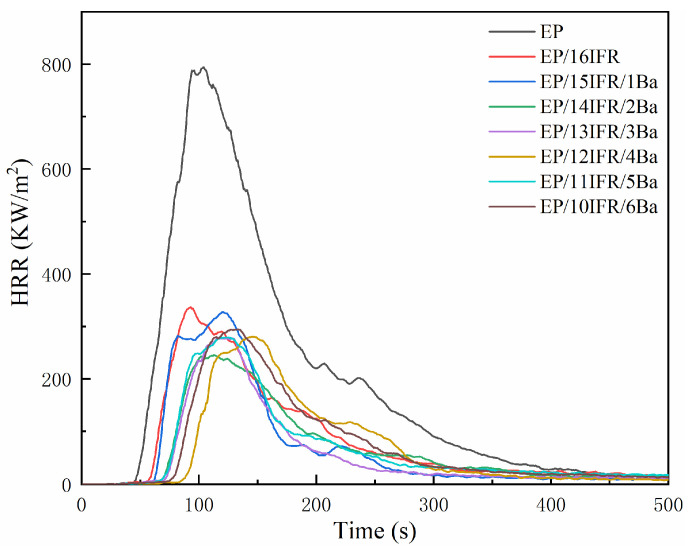
Curves of HRR of pure EP and EP composites with time.

**Figure 5:**
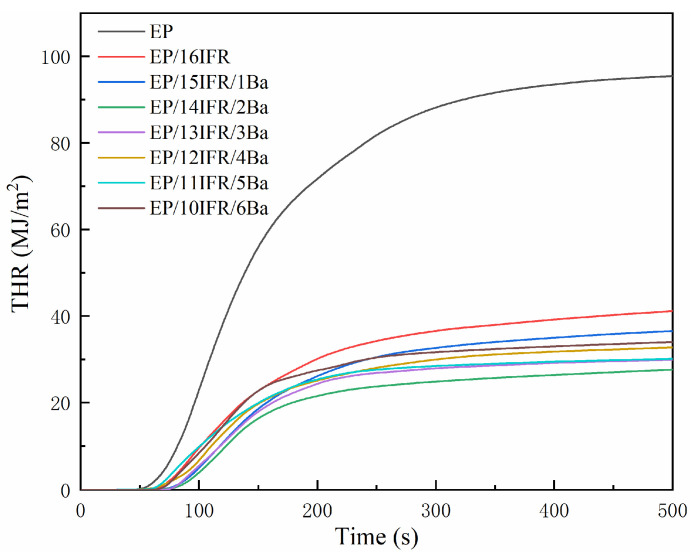
Curves of THR of pure EP and EP composites with time.

Curves of HRR and THR are displayed in Figure 4 and Figure 5, respectively. It can be recognized that pure EP has high flammability, showing the HRR is a single peak and changes rapidly over time, and the PHRR is as high as 794.09 kW/m^2^. The HRR curves of EP composites are analogous to those of pure EP. The addition of IFR slows down the heat release and decreases the PHRR value. After the addition of Pa-Ba, the value of PHRR decreases further, especially, the PHRR of EP/14IFR/2Ba reduces to the lowest level of 245.15 kW/m^2^, a decrease of 548.94 kW/m^2^, which is only 30.87% of that of pure EP. As displayed in Figure 5, pure EP has a THR of 95.45 MJ/m^2^. The THR of EP/IFR is remarkably lowered, which attains to 41.18 MJ/m^2^. After adding Pa-Ba, the value of THR decreases further, especially, the THR of EP/14IFR/2Ba drops to the lowest level of 27.16 MJ/m^2^, a decrease of 68.29 MJ/m^2^, which is only 28.45% of that of pure EP. From the HRR and THR curves, the addition of IFR and Pa-Ba can effectively reduce the PHRR and THR values, which attests that the flame retardancy of EP was improved [45].

**Figure 6:**
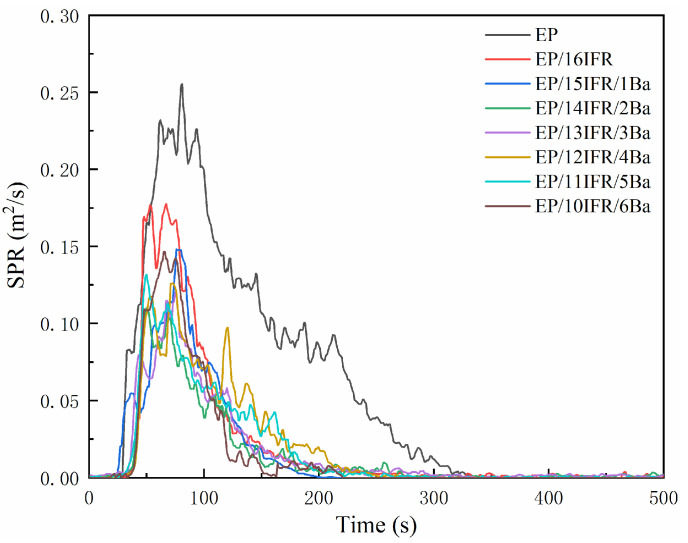
Curves of SPR of pure EP and EP composites with time.

**Figure 7:**
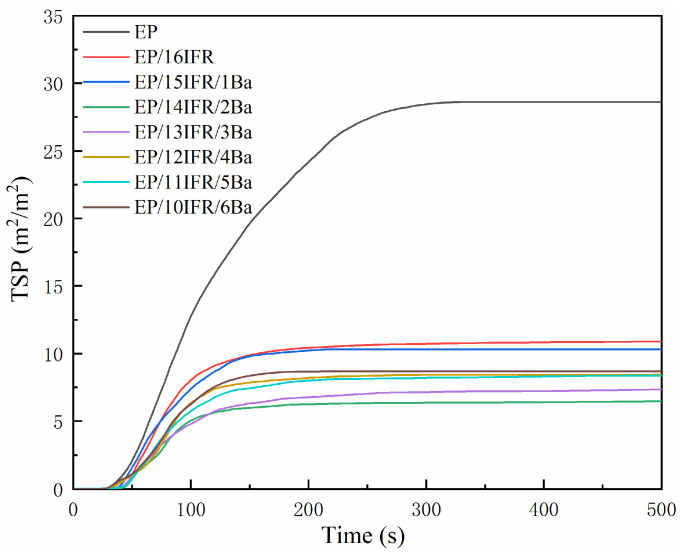
Curves of TSP of pure EP and EP composites with time.

Curves of SPR and TSP are exhibited in Figure 6 and Figure 7, respectively. As shown in Figure 6, the PSPR value of pure EP is 0.255 m^2^/s, which is much higher than that of EP composites. The addition of IFR and Pa-Ba slows down the smoke production of EP and decreases the PSPR value, especially, the PSPR of EP/14IFR/2Ba reduces to the lowest level of 0.109 m^2^/s, a decrease of 0.146 m^2^/s. From Figure 7, the TSP of EP composites decreases remarkably. Pure EP can produce smoke constantly and promptly, and the TSP is as high as 28.62 m^2^/m^2^. After adding IFR and Pa-Ba, the TSP of EP composites decreases dramatically, especially EP/14IFR/2Ba, the TSP value cuts down to the lowest level, which is only 6.48 m^2^/m^2^, a decrease of 22.14 m^2^/m^2^. From the SPR and TSP curves, the addition of IFR and Pa-Ba can availably decrease the SPR and TSP values and the total smoke volume of materials, which proves that EP composites have excellent smoke suppression performance. All these indicate that in the process of combustion, the existence of Pa-Ba can promote the cross-linking of IFR into carbon more efficiently, and the formed carbon layer can be used as an obstacle to block the transmission of heat and combustible gas, thus playing a better role of flame retardant.

### 3.4. Residual Char

**Figure 8:**
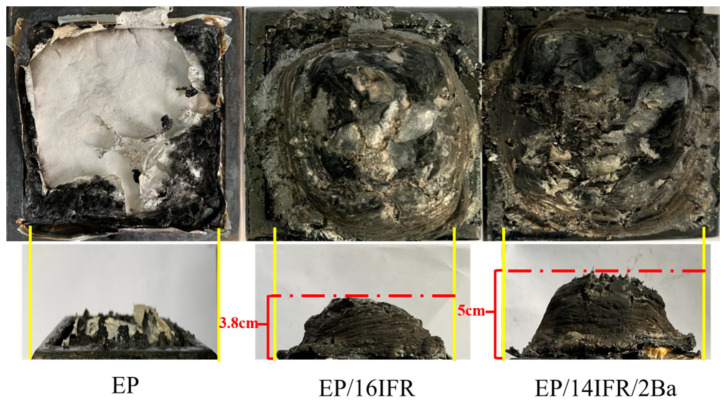
Digital photos of EP, EP/16IFR and EP/14IFR/2Ba after cone calorimeter testing.

Consecutive and dense residual char can have the effect of heat insulation and oxygen insulation, thus forestalling secondary combustion of the EP matrix. Figure 8 displays the digital photos of EP, EP/16IFR and EP/14IFR/2Ba after CCT. It is shown that pure EP is nearly burnt out and rarely produces residual char. Moreover, EP/16IFR has generated an expanded carbon layer, which is not compact enough and has a relatively low expansion height of 3.8 cm. However, the carbon layer generated by EP/14IFR/2Ba with the expansion height of 5.0 cm is relatively dense and consecutive, which means that the carbonization quality of EP/14IFR/2Ba is better than that of EP/16IFR.

**Figure 9:**
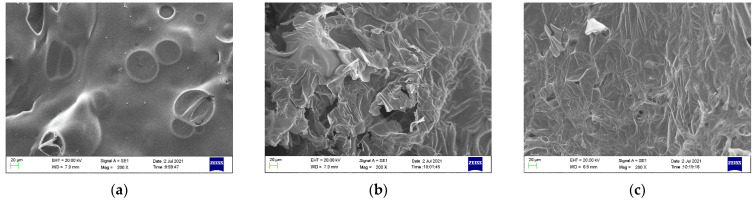
SEM of char residues of pure EP and EP composites, (**a**) EP, (**b**) EP/16IFR, and (**c**) EP/14IFR/2Ba.

Furthermore, the SEM of char residues is shown in Figure 9. For pure EP, although the surface is relatively smooth, numerous pores and cracks were found on the discontiguous residual char surface, making it impossible to delay the combustion of the underlying EP. In contrast, the continuance of the carbon layer of EP/16IFR is remarkably improved, with few pores produced and a compact and continuous carbon layer. Compared with EP/16IFR and pure EP, the addition of Pa-Ba forms a denser wrinkled carbon layer, which can be considered as a more effective protective barrier, not only to prevent molten droplets and the escape of combustible gas, but also have great effects on heat insulation and oxygen insulation.

**Figure 10:**
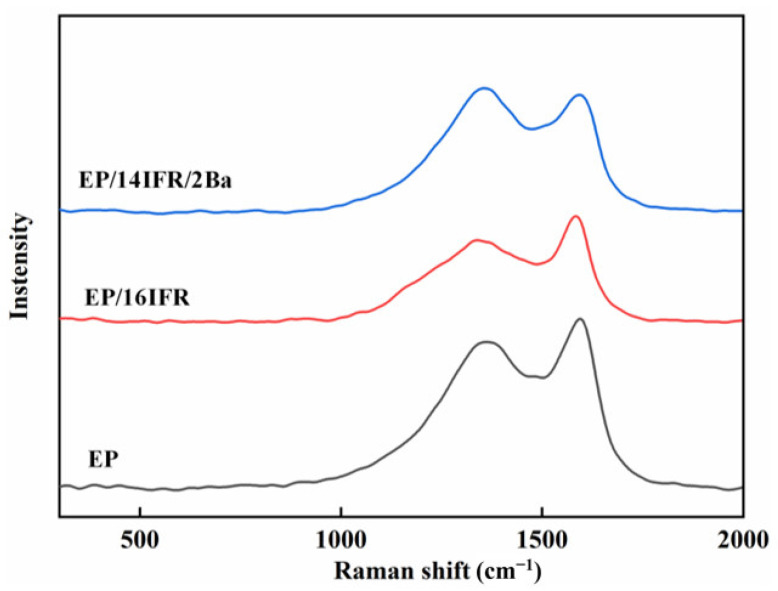
Raman spectra of EP, EP/16IFR and EP/14IFR/2Ba after combustion.

The residual chars after cone calorimeter testing are further researched as demonstrated in Figure 10. Based on the Raman spectra, we analyzed the characteristic peak of D-band and G-band in graphite, which appeared at 1348 and 1590 cm^−1^ in turn [46]. The D-band mainly corresponds to the defect of the graphitized layer, while the G-band is mainly equivalent to the ordered graphite layer. The ratio of R intensity of the D band to the G band (I_D_/I_G_) reflects the graphitization degree. Moreover, I_D_/I_G_ is inversely proportional to graphitization degree. The R values of pure EP, EP/16IFR and EP/14IFR/2Ba are 3.93, 3.52 and 3.15, respectively, indicating that the R value of EP/16IFR is lower and the degree of graphitization is higher compared with pure EP. After adding Pa-Ba, the R value decreases further, which means that the graphitization degree of the carbon layer is higher than that of EP/16IFR. Therefore, the carbon layer formed by EP/14IFR/2Ba after combustion is more orderly and dense, and its quality is better than that of the other two materials, which can be conducive to preventing the formation of cracks during and after combustion.

**Figure 11:**
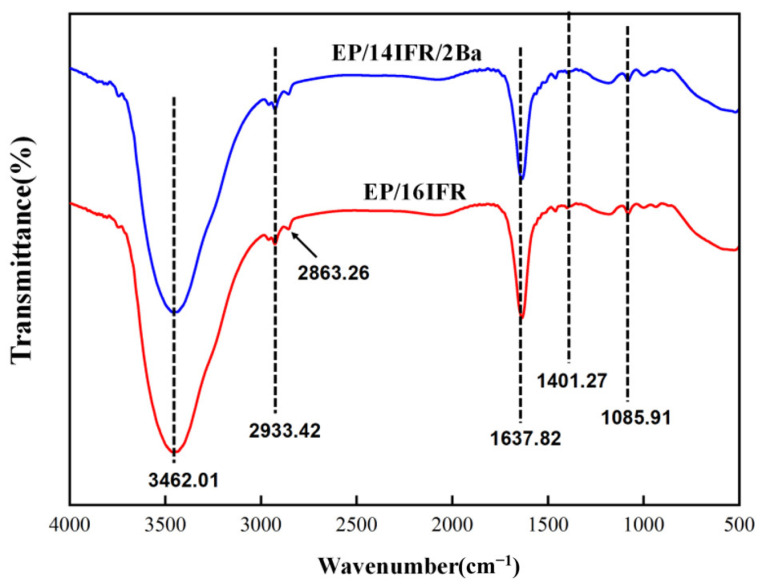
FTIR spectra of EP/16IFR and EP/14IFR/2Ba after combustion.

Curves of the FTIR spectra of residual chars are exhibited in Figure 11. As shown in EP/16IFR, 3462.01 cm^−1^ is associated with the O–H absorption, the appearance of the C–H telescopic vibration is shown in 2933.42 and 2863.26 cm^−1^, C=C meets the conditions of characteristic vibration peak at 1637.82 cm^−1^, the position of 1401.27 cm^−1^ is attributable to C–N stretching and N–H bending vibration absorption peak, and 1085.91 cm^−1^ is the characteristic band of P=O. It can be recognized from EP/14IFR/2Ba that there is no obvious difference between the two FTIR curves, demonstrating that the addition of Pa-Ba does not change the degradation products, only accelerating or delaying the reactions.

## 4. Conclusions

Barium phytate (Pa-Ba) was prepared from phytic acid and barium carbonate and characterized by FTIR and SEM. EP composites were produced by the addition of the synthesized Pa-Ba and IFR. The thermal stability was studied by TGA. EP/14IFR/2Ba has the highest residue of 25.90 wt%, which is much higher compared with pure EP (7.25 wt%). Afterwards, the flame retardancy was analyzed by LOI and CCT. The results show that Pa-Ba can cooperate with IFR to flame retardant EP, and EP/14IFR/2Ba has the highest LOI value of 30.7%. The PHRR value of EP/14IFR/2Ba decreases dramatically, from 794.09 kW/m^2^ to 245.15 kW/m^2^; meanwhile, the PSPR value reduces from 0.255 m^2^/s to 0.109 m^2^/s. From the residue char of EP composites after combustion, the expanded carbon layer generated by EP/14IFR/2Ba is dense and continuous, with a height of 5.0 cm. SEM and Raman spectroscopy were adopted to investigate the residue char further. They reveal that the carbonization quality of EP/14IFR/2Ba is better than that of other composites, which is conducive to preventing the formation of cracks during and after combustion.

The results demonstrate that Pa-Ba can be used as a carbon source, which can protect matrix degradation, prevent the escape of combustible gas, and have significant effects on heat insulation and oxygen insulation, thus forming more stable carbon layers. Meanwhile, Pa-Ba can improve the flame retardant efficiency of IFR in EP and reduce the total smoke volume of materials, so as to cooperate with IFR to improve the thermal stability, flame retardancy and smoke suppression performance of EP, thus playing a better role in reducing the probability of fire as well as expanding the applicable scope of EP.

## Figures and Tables

**Table 1:** The formulations of flame retarded EP.

Samples	EP (wt%)	Pa-Ba (wt%)	IFR (wt%)
EP	100	/	/
EP/16IFR ^a^	84	/	16
EP/15IFR/1Ba	84	1	15
EP/14IFR/2Ba	84	2	14
EP/13IFR/3Ba	84	3	13
EP/12IFR/4Ba	84	4	12
EP/11IFR/5Ba	84	5	11
EP/10IFR/6Ba	84	6	10

^a^ IFR: APP:PER = 3:1

**Table 2:** TGA data of pure EP and EP composites obtained in nitrogen atmosphere.

Samples	T_5 wt%_ (°C)	T_50 wt%_ (°C)	T_max_ (°C)	Residues at 800 °C (wt%)
EP	372	420	414	7.25
EP/16IFR	320	372	349	23.27
EP/15IFR/1Ba	315	377	342	23.08
EP/14IFR/2Ba	313	382	347	25.90
EP/13IFR/3Ba	325	379	349	25.22
EP/12IFR/4Ba	306	376	335	24.70
EP/11IFR/5Ba	314	379	346	25.06
EP/10IFR/6Ba	318	377	345	23.50

**Table 3:** LOI values of pure EP and EP composites.

Samples	Pa-Ba (wt%)	LOI (%)
EP	/	19.1
EP/16IFR	/	24.3
EP/15IFR/1Ba	1	26.7
EP/14IFR/2Ba	2	30.7
EP/13IFR/3Ba	3	29.0
EP/12IFR/4Ba	4	28.6
EP/11IFR/5Ba	5	27.5
EP/10IFR/6Ba	6	26.8

## Data Availability

All the data will be available to the readers.
